# Dysregulated TDP‐43 proteostasis perturbs excitability of spinal motor neurons during brainstem‐mediated fictive locomotion in zebrafish

**DOI:** 10.1111/dgd.12879

**Published:** 2023-07-20

**Authors:** Kazuhide Asakawa, Hiroshi Handa, Koichi Kawakami

**Affiliations:** ^1^ Laboratory of Molecular and Developmental Biology National Institute of Genetics Mishima Japan; ^2^ Department of Molecular Pharmacology, Center for Future Medical Research, Institute of Medical Science Tokyo Medical University Tokyo Japan; ^3^ The Graduate University for Advanced Studies (SOKENDAI) Mishima Japan

**Keywords:** ALS, calcium imaging, locomotion, spinal motor neuron, TDP‐43

## Abstract

Spinal motor neurons (SMNs) are the primary target of degeneration in amyotrophic lateral sclerosis (ALS). Degenerating motor neurons accumulate cytoplasmic TAR DNA‐binding protein 43 (TDP‐43) aggregates in most ALS cases. This SMN pathology can occur without mutation in the coding sequence of the TDP‐43‐encoding gene, *TARDBP*. Whether and how wild‐type TDP‐43 drives pathological changes in SMNs in vivo remains largely unexplored. In this study, we develop a two‐photon calcium imaging setup in which tactile‐evoked neural responses of motor neurons in the brainstem and spinal cord can be monitored using the calcium indicator GCaMP. We devise a piezo‐assisted tactile stimulator that reproducibly evokes a brainstem descending neuron upon tactile stimulation of the head. A direct comparison between caudal primary motor neurons (CaPs) with or without TDP‐43 overexpression in contiguous spinal segments demonstrates that CaPs overexpressing TDP‐43 display attenuated Ca^2+^ transients during fictive escape locomotion evoked by the tactile stimulation. These results show that excessive amounts of TDP‐43 protein reduce the neuronal excitability of SMNs and potentially contribute to asymptomatic pathological lesions of SMNs and movement disorders in patients with ALS.

## Graphical abstract

The mechanism underlying neurotoxicity of TDP‐43 in ALS has been extensively studied but remains poorly understood. Using two‐photon calcium imaging of the neural circuitry for the brainstem‐mediated escape locomotion in zebrafish, we demonstrate that the excessive amount of TDP‐43 protein perturbs the excitability of spinal motor neurons. This TDP‐43‐dependent motor neuron pathology potentially contributes to movement disorders in ALS.

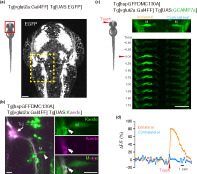

## 1 INTRODUCTION

Amyotrophic lateral sclerosis (ALS) is a neurodegenerative disorder characterized by the progressive loss of both upper and lower motor neurons. A hallmark of ALS is the deposition of cytoplasmic aggregates of TAR DNA‐binding protein 43 (TDP‐43) in degenerating motor neurons (Arai et al., 2006; Neumann et al., 2006). Cytoplasmic TDP‐43 aggregates have been detected in almost all cases of sporadic ALS (Mackenzie et al., 2007), accounting for more than 90% of all ALS cases (Taylor et al., 2016). The remaining 10% of ALS cases are heritable forms of ALS, in which mutations in the *TARDBP* gene encoding TDP‐43 are identifiable at a certain frequency.

An important but largely unanswered question in ALS is whether and how dysregulation of wild‐type TDP‐43 affects the function of motor neurons in sporadic ALS cases, in which mutations in the coding sequence of the *TARDBP* gene are absent. TDP‐43 is an RNA/DNA‐binding protein with a wide range of target nucleic acids, and its dysfunction likely has a widespread impact on the cellular transcriptome and proteome (Acharya et al., 2006; Li et al., 2022; Ou et al., 1995; Polymenidou et al., 2011; Sephton et al., 2011; Tollervey et al., 2011; Xiao et al., 2011). Recent studies have revealed that a significant consequence of the loss of TDP‐43 function is the occurrence of splicing abnormalities, leading to erroneous incorporation of intronic sequences into mature mRNA (Brown et al., 2022; Koike et al., 2023; Ling et al., 2015; Ma et al., 2022; Mehta et al., 2023). Overexpression of wild‐type TDP‐43 can also be toxic in various models (Asakawa et al., 2020; Barmada et al., 2010; Estes et al., 2011; Johnson et al., 2008; Kabashi et al., 2010; Wils et al., 2010; Xu et al., 2010). Excessive amounts of wild‐type TDP‐43 impair splicing accuracy. For example, overexpression of wild‐type TDP‐43 in human cultured cells causes exon skipping in the pre‐mRNA of the cystic fibrosis transmembrane conductance regulator (CFTR) protein, resulting in an inactive translation product (Buratti et al., 2001). The effect of the molecular‐level dysfunction of wild‐type TDP‐43 on motor neuron excitability is poorly understood. In the mouse model, ectopic expression of human nuclear localization sequence‐deficient TDP‐43 (TDP‐43ΔNLS) results in hyperexcitability of layer V excitatory neurons in the cortex, while ectopic expression of human wild‐type TDP‐43 is tolerated with only subtle electrophysiological changes in the cortex (Dyer et al., 2021). In the zebrafish model, injection of mRNA encoding a human mutant TDP‐43 at the one‐cell stage blastula results in the augmentation of spinal cord glutamatergic synaptic currents in primary motor neurons in the spinal cord; however, overexpression of the wild‐type TDP‐43 protein has not been reported to result in a discernible phenotype (Petel Legare et al., 2019).

In this study, we investigated the effects of excessive TDP‐43 protein levels on the neural activity of spinal motor neurons (SMNs) in the context of escape locomotion in zebrafish larvae. Larval zebrafish serve as a valuable model for studying motor neuron function because of their translucent bodies, allowing for direct visualization of motor neurons in the spinal cord at single‐cell resolution. We utilized the genetically encoded calcium imaging probe GCaMP7a to monitor the neural activity of SMNs during tactile‐induced fictive escape locomotion and found that SMNs overexpressing fluorescently tagged wild‐type TDP‐43 displayed reduced calcium transients during fictive escape locomotion. These results suggest that excessive amounts of TDP‐43 protein reduce the neuronal excitability of SMNs during locomotion, potentially contributing to movement disorders in ALS.

## 2 MATERIALS AND METHODS

### 2.1 Fish husbandry

This study was performed in accordance with the Guide for the Care and Use of Laboratory Animals of the Institutional Animal Care and Use Committee of the National Institute of Genetics (NIG, Japan, approval numbers: 26‐4 and R5‐1). Fish were raised under a 12:12 h light/dark cycle during the first five days after birth.

### 2.2 Tg[vglut2a:Gal4] line

For generation of the bacterial artificial chromosome (BAC) transgene Tg[vglut2a:Gal4], the BAC clone DKEY‐145P24 was used. The *hsp70l* promoter (650 bp)‐Ga4FF‐polyA‐Km^r^ cassette was introduced downstream of the vglut2a 5′‐untranslated region in the vglut2a‐BAC DNA in SW102 cells (Asakawa & Kawakami, 2018; Warming et al., 2005). For transgenesis of the engineered vglut2a‐BAC, the iTol2‐amp cassette (Suster et al., 2011) was amplified by PCR with the primer pair iTol2A‐IndB5‐f (5′‐cga gcc gga agc ata aag tgt aaa gcc tgg gg tgc cta atg agt gag cta CCC TGC TCG AGC CGG GCC C‐3′) and iTol2A‐IndB5‐r (5′‐ggt ttc ccg act gga aag cgg gca gtg agc gca acg caa tta atg tga gtA TTA TGA TCC TCT AGA TCA GAT CT‐3′), where the lower‐ and uppercase letters indicate the pIndigoBAC‐5 sequences for homologous recombination and iTol2‐amp annealing sequences, respectively, and introduced into the backbone (pIndigoBAC‐5, GenBank Accession; EU140754). The resulting vglut2a‐BAC carrying the iTol2‐amp cassette was purified and injected into one‐cell stage zebrafish embryos with Tol2 transposase mRNA (Asakawa et al., 2013).

### 2.3 Kaede photoconversion

The trigeminal ganglion of Tg[vglut2a‐Gal4]; Tg[vglut2a:Gal4]; Tg[UAS:Kaede] fish embedded in 1% low‐melt agarose was irradiated with a laser at a wavelength of 405 nm using a confocal laser scanning microscope (FV‐1200D, Olympus) at 44 h post‐fertilization (hpf ).

### 2.4 Calcium imaging and data analysis

Two‐photon calcium imaging was performed using an LSM 7MP microscope (Zeiss) equipped with Mai Tai eHPDS lasers (Spectra‐Physics). To minimize body movement during imaging, fish at 48 hpf were treated with a neuromuscular junction blocker, d‐tubocurarine (100 μM, Sigma; T2379), after their tail tips were cut and embedded in 2% low‐melting agarose (Ronza). The agarose around the head region was removed for tactile stimulation using a tungsten needle (NILACO) attached to an electrode holder (H‐12, NARISHIGE) controlled by a piezo actuator (MC‐140L, MESS‐TEK), piezo‐driver (M‐2674, MESS‐TEK), and function generator (DF‐1906). Calcium imaging was performed in a single *z*‐section that included somas of interest. Images were acquired at 2.5 Hz. Calcium signals in the soma areas were analyzed using Fiji/ImageJ software. The basal level of the GCaMP7 signal (F_0_) was defined as the mean intensity of the GCaMP7 signal about 5 s before the tactile stimulation. ∆F was calculated by subtracting the F_0_ signal from the GCaMP7 signal. Results were obtained from 38 trials of 14 CaPs with nuclear mRFP1‐TDP‐43z and 10 CaPs without nuclear mRFP1‐TDP‐43z in three animals. The maximum ∆F/F values between CaPs with or without nuclear mRFP1‐TDP‐43z signal were analyzed by non‐paired and paired *t*‐tests using GraphPad Prism 9 software.

## 3 RESULTS AND DISCUSSION

**FIGURE 1:**
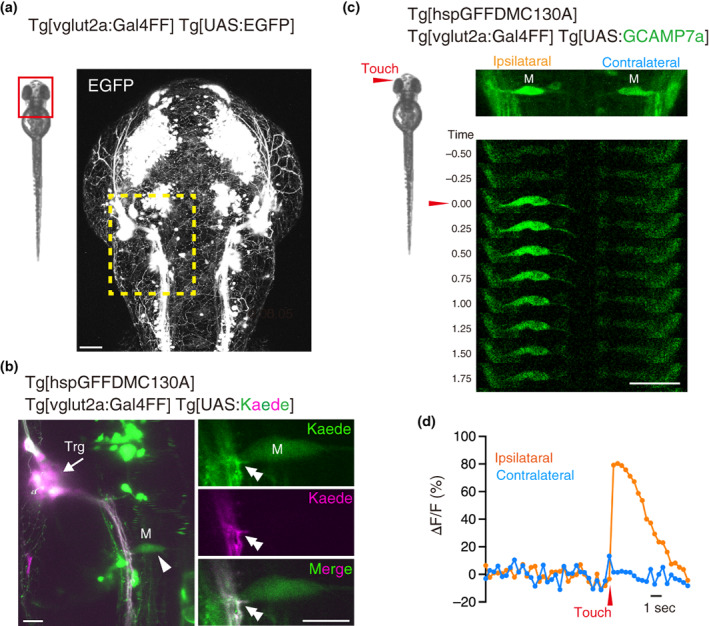
Anatomical and functional connections between trigeminal sensory neurons and Mauthner cells in zebrafish larvae. (a) Dorsal view of the head region of Tg[vglut2a:Gal4FF]; Tg[UAS:EGFP] fish at 48 hpf. The dashed rectangle indicates the area corresponding to the brainstem region analyzed in (b). (b) Left panel: Kaede expressed in the trigeminal sensory neurons is photoconverted by illuminating the trigeminal ganglion region (Trg, arrow) with a laser light with a 405 nm wavelength (magenta). Afferent axons are projected towards a Mauthner cell (arrowhead) highlighted in magenta. Right panels: A single confocal section of the contact site (double arrowheads) of the afferent axons of trigeminal sensory neurons and Mauthner cells. (c) Calcium imaging of the Mauthner cells before and after tactile stimulation. (d) ∆F/F of the neural response of Mauthner cells in (c). Scale bars indicate 50 μm (c) or 20 μm (a, b).

To investigate the effect of dysregulated TDP‐43 proteostasis on SMNs in vivo, we focused on the brainstem‐mediated escape locomotor circuit that assembles during the larval stage of zebrafish. Zebrafish develops neural circuits for robust escape locomotion upon the perception of tactile stimuli by the time of hatching (approximately 48 hpf) (Saint‐Amant & Drapeau, 1998). Tactile stimuli in the head region are perceived through the peripheral sensory arbors of the trigeminal sensory neurons that tile the body surface (Sagasti et al., 2005). The central axons of the trigeminal sensory neurons extend along the descending longitudinal fiber pathway and contact the brainstem descending neurons, including the Mauthner cell, which is activated during the escape response (Kimmel et al., 1990). We explored the anatomical relationship between the trigeminal sensory neurons and the Mauthner cell at 48 hpf by simultaneously visualizing the central axon of the trigeminal sensory neurons and the Mauthner cell by combining a BAC transgenic Gal4 line for vglut2a (L‐glutamate transmembrane transporter) (Figure 1a), the Gal4 driver for the Mauthner cell Tg[hspGFFDMC130A] (Asakawa et al., 2008; Pujol‐Marti et al., 2012), and Tg[UAS:Kaede] (Scott et al., 2007). The central axons of the trigeminal sensory neurons were highlighted with red fluorescence by the photoconversion of Kaede, which was expressed in the trigeminal ganglion, with a laser light with a 405 nm wavelength in the Tg[vglut2a‐Gal4]; Tg[vglut2a:Gal4]; Tg[UAS:Kaede] triple transgenic fish at 48 hpf (Figure 1b). The central axons of the trigeminal sensory neurons descended in the vicinity of the lateral region of the Mauthner cell soma (Figure 1b), suggesting neuronal contact between the bundle of central axons of the trigeminal sensory neurons and the Mauthner cell, consistent with previous observations (Kimmel et al., 1990). To establish a functional connection between trigeminal sensory neurons and Mauthner cells at 48 hpf, we set up a system that enables the tactile‐evoked neuronal activity of the Mauthner cell to be visualized by calcium imaging using two‐photon microscopy. The Tg[hspGFFDMC130A] driver was crossed with Tg[UAS:GCaMP7a] (Muto et al., 2013) to express the calcium indicator GCaMP7a in Mauthner cells. Tg[hspGFFDMC130A]; Tg[vglut2a:Gal4]; Tg[UAS:GCaMP7a] triple transgenic fish at 48 hpf were briefly treated with a neuromuscular blocker, embedded upright in agarose, and subjected to calcium imaging under a two‐photon microscope. We set up a piezo‐controlled tungsten needle for controlled application of tactile stimuli. A single *z*‐section including the bilateral Mauthner cells was scanned using a two‐photon laser at 2.5 Hz (Figure 1c). When a tactile stimulus was applied to the head skin near the eye during imaging, the Mauthner cell ipsilateral to the stimulus strongly increased GCaMP7a fluorescence, whereas the contralateral Mauthner cell displayed only a minimal increase in the GCaMP7a signal (Figure 1c,d). These observations demonstrate that trigeminal sensory neurons and ipsilateral Mauthner cell establish functional neural connections by 48 hpf and likely contribute to touch‐evoked escape locomotion at this stage.

**FIGURE 2:**
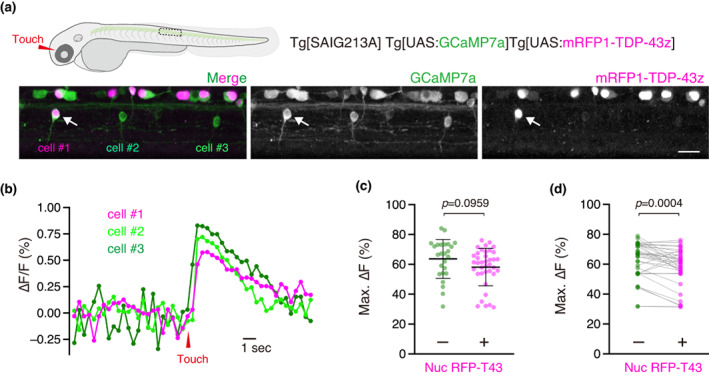
Calcium imaging of the CaPs overexpressing mRFP1‐TDP‐43z. (a) Lateral view of the spinal cord of Tg[SAIG213A]; Tg[UAS:GCaMP7a]; Tg[UAS:mRFP1‐TDP‐43z] fish at 48 hpf. Among the three CaPs shown, cell #1 (arrow) displayed the nuclear mRFP1‐TDP‐43z signal, whereas cell #2 and cell #3 did not. (b) ∆F/F of the three CaPs before and after tactile stimulation of the head. (c, d) Comparison of maximum ∆F/F values between CaPs with or without nuclear mRFP1‐TDP‐43z signal (Nuc RFP‐T43) using non‐paired (c) and paired *t*‐tests (d). Results were obtained from 38 trials of 14 CaPs with nuclear mRFP1‐TDP‐43z and 10 CaPs without nuclear mRFP1‐TDP‐43z in three animals. The scale bar indicates 20 μm.

Having established a tactile stimulation setup under a two‐photon microscope, we investigated the effect of wild‐type TDP‐43 overexpression on the excitability of SMNs during tactile‐induced fictive escape locomotion. We crossed the Tg[SAIG213A] Gal4 driver with Tg[UAS:GCaMP7a] to drive GCaMP7a expression in caudal primary motor neurons (CaPs), which innervate the ventral third of the myotome (Myers et al., 1986). To overexpress TDP‐43 in CaPs, we used the Tg[UAS:mRFP1‐TDP‐43z] line, which expresses mRFP1‐tagged zebrafish TDP‐43 in a Gal4‐dependent manner. This mRFP1‐tagged TDP‐43 primarily localizes to the nuclei of CaPs without discernable deposition of cytoplasmic mRFP1‐TDP‐43z aggregates (Asakawa et al., 2020). While in Tg[SAIG213A]; Tg[UAS:mRFP1‐TDP‐43z]; Tg[UAS:GCaMP7a] triple transgenic fish GcaMP7a was expressed in almost all CaPs, mRFP1‐TDP‐43z was not detectable in some of these CaPs, owing to transgene silencing (Figure 2a). We took advantage of the variegated expression of mRFP1‐TDP‐43z and used CaPs expressing GcaMP7a without a nuclear mRFP1‐TDP‐43z signal as an internal control. We set a region of interest in the spinal cord of laterally positioned Tg[SAIG213A]; Tg[UAS:mRFP1‐TDP‐43z]; Tg[UAS:GCaMP7a] fish embedded in agarose and a single *z*‐section that sliced two to three CaPs expressing GCaMP7a, at least one of which did not contain the nuclear mRFP1‐TDP‐43z signal. When a tactile stimulus was applied to the head, the GCaMP7a signals of the CaPs robustly increased and persisted for approximately 10 s, regardless of the presence of the nuclear mRFP1‐TDP‐43z signal (Figure 2b). While a collective comparison of the maximum ∆F/F signals of GCaMP7a from all trials did not reveal a significant effect of mRFP1‐TDP‐43z expression (Figure 2c), pairwise comparisons within adjacent spinal segments confirmed that mRFP1‐TDP‐43z overexpression significantly attenuated the maximum ∆F/F signal of the CaPs (Figure 2d). Notably, the CaPs on both sides of the spinal hemisegments were activatable in response to lateralized tactile stimulation, suggesting that the ∆F/F signal of GCaMP7a in the CaPs reflects not only neural excitation via a contralateral monosynaptic connection by the Mauthner cell (Jontes et al., 2000), but also burst activity during fictive escape swimming. Taken together, these observations indicate that TDP‐43 overexpression reduced the neural excitability of SMNs during locomotion.

In the present study, we performed two‐photon calcium imaging of the neural activity of the escape locomotor circuit and found that the overexpression of mRFP1‐tagged wild‐type TDP‐43 reduced the neural excitability of the SMNs during tactile‐induced fictive escape locomotion. Reduced neural excitability has been associated with the overexpression of TDP‐43 carrying ALS mutations in several animal models, but not with the overexpression of wild‐type TDP‐43 (Arnold et al., 2013; Dyer et al., 2021; Petel Legare et al., 2019). Direct comparison of the neural activity of SMNs with or without TDP‐43 overexpression at a single‐cell resolution clearly showed that wild‐type TDP‐43 affects the excitability of SMNs when in excess.

By definition, a decrease in ∆F/F indicates a reduction in the peak intracellular Ca^2+^ level by neural excitation when an excessive amount of TDP‐43 is present in the SMNs. Several possible mechanisms may explain this decrease in ∆F/F. The first is that the influx of extracellular Ca^2+^ into the cytosol upon plasma membrane depolarization is attenuated in CaPs overexpressing TDP‐43. TDP‐43 is known to regulate the transcription of voltage‐gated Ca^2+^ channels (CaV1.2) in the pancreatic β‐cell line MIN6, and TDP‐43 depletion downregulates CaV1.2 at the transcriptional level (Araki et al., 2019). The physical association of TDP‐43 with CaV1.2 mRNA was shown in the mouse brain by cross‐linking and immunoprecipitation experiments (Polymenidou et al., 2011). Furthermore, locomotor deficits in flies lacking neuronal TDP‐43 are rescued by exogenous expression of voltage‐gated calcium channels (Chang et al., 2014). Locomotor defects in zebrafish expressing TDP‐43 with the ALS mutation G348C were restored by treatment with L‐type calcium channel agonists (Armstrong & Drapeau, 2013). These observations support the hypothesis that reduced Ca^2+^ influx via CaV1.2 causes attenuated neuronal excitability in SMNs overexpressing TDP‐43. Another possibility that is not mutually exclusive with the first is that the insufficient release of Ca^2+^ from the endoplasmic reticulum reduces the level of cytosolic Ca^2+^ upon neural excitation. Overexpression, but not reduction of function, of TDP‐43 reduces the binding of VAPB to PTPIP51, which is essential for mitochondria‐associated membrane (MAM) function, in both transfected cells and transgenic mice (Stoica et al., 2014). Given the critical role of MAM in intracellular Ca^2+^ homeostasis, TDP‐43 overexpression‐dependent MAM disruption may cause a reduction in ∆F/F. It is possible that other cellular modules involved in Ca^2+^ homeostasis and/or motor neuron excitability are affected by mRFP1‐TDP‐43z overexpression.

A limitation of this study is that we could not quantitatively determine the extent to which mRFP1‐TDP‐43z is overexpressed in CaPs in relation to endogenous TDP‐43 proteins expressed from two paralogous genes for TDP‐43 in zebrafish, *tardbp* and *tardbpl*. However, mRFP1‐TDP‐43z overexpression by the Tg[SAIG213A] driver did not lead to the deposition of mRFP1‐TDP‐43z aggregates in the cytoplasm of CaPs (Asakawa et al., 2020), showing that Tg[SAIG213A]‐driven mRFP1‐TDP‐43z overexpression is enough to cause cytotoxicity, but not sufficient to induce the formation of its cytoplasmic aggregates. As we have previously shown that the Tg[SAIG213A]‐driven mRFP1‐TDP‐43z overexpression halts the axon outgrowth of CaPs (Asakawa et al., 2020), any causal relationship between the axon outgrowth defects and reduced neural excitability upon mRFP1‐TDP‐43z overexpression should be explored in the future. We posit that the reduced motor unit size and diminished neural excitability caused by mRFP1‐TDP‐43z overexpression might correspond to asymptomatic pathological lesions in SMNs that occur before the accumulation of cytoplasmic TDP‐43 aggregates and later contribute to the manifestation of motor dysfunction in ALS.

## AUTHOR CONTRIBUTIONS

Kazuhide Asakawa conceived the research and designed and performed the experiments. Kazuhide Asakawa and Koichi Kawakami analyzed the data. Kazuhide Asakawa, Hiroshi Handa, and Koichi Kawakami wrote the manuscript.
